# Characteristics of undernourished older medical patients and the identification of predictors for undernutrition status

**DOI:** 10.1186/1475-2891-6-37

**Published:** 2007-11-02

**Authors:** Ilana Feldblum, Larisa German, Hana Castel, Ilana Harman-Boehm, Natalya Bilenko, Miruna Eisinger, Drora Fraser, Danit R Shahar

**Affiliations:** 1 The S. Daniel Abraham International Center for Health and Nutrition, Ben-Gurion University of the Negev, Beer-Sheva, Israel; 2 The multidisciplinary center for gerontology and aging research, Ben-Gurion University of the Negev, Beer-Sheva, Israel; 3 Department of Internal Medicine C, Soroka University Medical Center and the Faculty of Health Sciences, Ben-Gurion University of the Negev, Beer-Sheva, Israel; 4 Department of Internal Medicine F, Soroka University Medical Center Beer-Sheva, Israel

## Abstract

**Background:**

Undernutrition among older people is a continuing source of concern, particularly among acutely hospitalized patients. The purpose of the current study is to compare malnourished elderly patients with those at nutritional risk and identify factors contributing to the variability between the groups.

**Methods:**

The study was carried out at the Soroka University Medical Center in the south of Israel. From September 2003 through December 2004, all patients 65 years-of-age or older admitted to any of the internal medicine departments, were screened within 72 hours of admission to determine nutritional status using the short version of the Mini Nutritional Assessment (MNA-SF). Patients at nutritional risk were entered the study and were divided into malnourished or 'at risk' based on the full version of the MNA. Data regarding medical, nutritional, functional, and emotional status were obtained by trained interviewers.

**Results:**

Two hundred fifty-nine elderly patients, 43.6% men, participated in the study; 18.5% were identified as malnourished and 81.5% were at risk for malnutrition according to the MNA. The malnourished group was less educated, had a higher depression score and lower cognitive and physical functioning. Higher prevalence of chewing problems, nausea, and vomiting was detected among malnourished patients. There was no difference between the groups in health status indicators except for subjective health evaluation which was poorer among the malnourished group. Lower dietary score indicating lower intake of vegetables fruits and fluid, poor appetite and difficulties in eating distinguished between malnourished and at-risk populations with the highest sensitivity and specificity as compare with the anthropometric, global, and self-assessment of nutritional status parts of the MNA. In a multivariate analysis, lower cognitive function, education <12 years and chewing problems were all risk factors for malnutrition.

**Conclusion:**

Our study indicates that low food consumption as well as poor appetite and chewing problems are associated with the development of malnutrition. Given the critical importance of nutritional status in the hospitalized elderly, further intervention trials are required to determine the best intervention strategies to overcome these problems.

## Background

Undernutrition among older people is a continuing source of concern [1], particularly among acutely hospitalized patients [2]. Undernourished elderly have longer periods of illness, longer hospital stays [3,4], higher rates of infection [5,6], delayed wound healing [7], reduced appetite [8,9], and increased mortality rates [10].

Data from previous studies indicate that up to 55% of elderly hospitalized patients are undernourished or malnourished on admission [11,12]. Both undernutrition and malnutrition are associated with poorer recovery in a broad range of patients and conditions [1-10]. Undernutrition adversely affects both lifespan and quality of life in community-dwelling older people [13-15] and is a critical determinant of outcomes among aging in-patients, influencing the outcome of the hospitalization [1,15] and correlating with morbidity [16-21] and mortality [22-24] in this population. The risk for nutritional deterioration, is greater than the prevalence of actual malnourishment reported [25,26].

In Israel, nutritional assessment of institutionalized [27,28,21] and home-dwelling [29,30] elderly has been performed. Studies conducted in the Negev, the southern part of Israel, indicate that among community dwelling elderly, the energy intake was below 78% of the RDA for men and 70% for women [30]. Data regarding the nutritional status of acutely hospitalized patients are sparse. In a study conducted by our group, we found the prevalence of nutritional risk among older patients on admission to a medical ward to be 38.7% [9]. In previous studies that were conducted in Israel [9,27-30] and other places [1-8,10], no attention was given to the different health characteristics of malnourished subjects and those at nutritional risk.

The importance of distinguishing nutritional risk from actual malnutrition lies in the difference between these conditions in terms of outcomes. While nutritional risk, if identified, is amenable to intervention which may reverse its course, actual malnutrition is more likely to persist and contribute to poorer outcome.

This study compares malnourished elderly patients with those at nutritional risk and identifies factors contributing to the variability between the groups.

## Methods

The study was carried out at Soroka University Medical Center (a 1000-bed university-affiliated acute care hospital) in the south of Israel. From September 2003 through December 2004, all patients 65 years-of-age or older admitted to any of the internal medicine departments were screened within 72 hours of admission to determine nutritional status. Screening was performed by the short version of the Mini Nutritional Assessment (MNA-sf) [31], a simple, validated screening tool for nutritional risk in elderly persons or by a history of weight loss ≥10% of their body weight in the 6 months prior to their admission. Weight loss has been shown in several studies to be the most important predictor for nutritional deterioration [19] (weight loss in the last 3 months is part of the nutritional evaluation of the MNA), thus it was included as an independent screening question.

**Figure 1:**
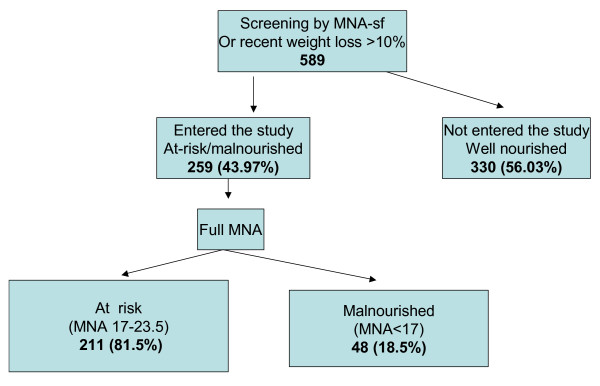
Flow chart for study participant selection.

Subjects who were identified as being at nutritional risk by one of these parameters were entered into the study (Figure 1). Exclusion criteria included cancer, inability to be interviewed, or unwillingness to sign an informed consent.

The protocol of the study was approved by the ethics committee of Soroka University Medical Center. An informed consent form was signed by all participants.

Participants recruited into the study were interviewed by trained interviewers. The data collected included demographic information as well as nutritional, health, cognitive, emotional, and functional evaluation.

### Demographic data

Demographic variables, including sex, age, marital status, country of origin, education, and living arrangements, were obtained from the patient and from the hospital admission form.

### Nutritional assessment

The short version of the MNA that was used for screening was used to complete the full version. The MNA consists of four parts:

1. Anthropometric measurements – Questions 1–4 include current body mass index (BMI), mid-arm circumference (MAC), calf circumference (CC), and weight loss in the last 3 months.

2. Global assessment – Questions 5–10 include living arrangements, number of prescribed medications, psychological stress in the last 3 months, mobility, neuropsychological problems, and pressure sores.

3. Dietary assessment – Questions 11–16 include number of full meals per day, protein intake, fruit and vegetables intake (over 2 portions per day), decrease in food intake in the last 3 months, fluid intake per day, and the ability to eat alone.

4. Subjective assessment – Questions 17 and 18 include subjective assessment of the participant's nutritional and health status.

The total score of the MNA distinguished between patients at nutritional risk (MNA score between 17 and 23.5) and patients with protein-calorie malnutrition (MNA score < 17) [32,33]. Participants with an MNA score >23.5 who had lost more than 10% of their body weight in the 6 months prior to the study period, were entered into the at risk group (Figure 1).

To assess specific eating problems we used selected questions from the Nutrition Risk Index (NRI) questionnaire [34] that were analyzed separately. The questions related to swallowing and chewing problems, vomiting, constipation and diarrhea, and use of special diets were included.

### Clinical data

Clinical data obtained from the patients' charts included biochemical measurements relevant to nutritional status such as albumin, total lymphocyte count (TLC), hemoglobin, WBC, total cholesterol, and transferrin. The tests were performed at the central chemistry lab of Soroka University Medical Center using standard methods.

### Functional measurements

Cognitive status was determined using the Folstein Mini Mental State Examination (MMSE) [35]. The MMSE score ranges from 0 to 30; a score of less than 24 indicates cognitive impairment. Depressive symptoms were assessed using the short form of the Geriatric Depression Screening Scale Short Form (GDS-sf) [36]. The GDS-sf score ranges from 0 to 15. A cutoff score of 5 or greater indicates depressive symptoms. Functional status was assessed using the modified Barthel Index [37], based on basic activities of daily living (ADL). The score ranges from 0 to 100, where 0 represents being totally dependent and 100 totally independent. The interviewers were trained to use these forms.

### Statistical analyses

Statistical analyses were conducted using SPSS for Windows version 14. Baseline characteristics were recorded and entered into a data management program. The data were edited and the distribution of all the relevant variables was evaluated for normality. Comparison between malnutrition and at risk for malnutrition based on the full version of the MNA was conducted using *t*-tests for continuous variables and χ^2 ^for categorical variables. To assess the predictive abilities of specific MNA topics (full version) to the total score, we assessed the area under the curve using a receiver operating characteristic curve (ROC). Sensitivity and specificity were calculated based on the ROC curve. The sensitivity of the model was defined as the percentage of malnourished participants who were correctly identified by the test. Alternately, specificity was defined as the percentage of participants at nutritional risk who were correctly identified. The sum of sensitivity and specificity defined the validity (area under the curve) of the specific MNA topics including anthropometric, dietary, global, and self-assessment.

Multivariate analysis was conducted using a logistic regression model in which nutritional status, at risk or malnutrition, was used as the dependent variable.

## Results

A total of 259 patients entered the study, 43.6% men, with a mean age of 75 years. Within the study group, 18.5% were identified as malnourished and 81.5% were at nutritional risk. The mean MNA score was 19.5. Baseline characteristics of the study population by nutritional status are shown in Table 1. Malnourished participants had a significantly lower level of education and BMI, and lower marriage rates compared with those at risk for malnutrition. Almost 64% of the study population immigrated from Europe/America, 34% immigrated from Africa/Asia, and the rest were born in Israel. Compared with the at risk participants, a higher percent of immigrants from Africa/Asia were included in the malnourished group. There was no difference in age, sex, and living arrangement between the two groups. Examination of functional variables revealed significant differences between the two groups in several functional abilities. Higher rates of functional disability were found among the malnourished group. This group also had a higher depression score (indicating more depressive symptoms), and a lower cognitive function score (indicating decreased cognitive ability). Interestingly, there was no difference between the groups in number of diagnosed diseases, number of prescribed medications, number of hospitalizations in the year before the study, number of family and specialist doctor visits, and duration of hospitalization.

**Table 1:** Characteristics of the Study Population by Nutritional Status.

Variable	Malnourished	Risk of malnutrition	Total	P value
No. of participants (%)	48 (18.5%)	211 (81.5%)	259	
Age (Mean ± SD)	75.0+5.5	75.2+5.8	75.2+5.8	0.87
Sex (%)				
Men	43.8%	43.6%	43.6%	0.99
Women	56.3%	56.4%	56.4%	
Education ≤12 years (%)	89.6%	65.4%	69.9%	0.001
Living alone (%)	39.6%	36%	36.7%	0.64
BMI (Mean ± SD)	25.6+5.9	27.8+5.0	27.4+5.2	0.019
Family status (%)				
Married	31.3%	49.8%	46.3%	0.06
Other	4.2%	4.3%	4.2%	
Widowed	64.6%	46%	49.4%	
Number of diagnosed diseases (Mean ± SD)	7.7 ± 2.8	7.35 ± 2.5	7.4 ± 2.6	0.36
Country of Origin (%)				
Europe/America	52.1%	66.4%	63.7%	0.05
Africa/Asia	47.9%	30.8%	34%	
Israel	0%	2.8%	2.3%	
Number of medications (Mean ± SD)	6.3 ± 2.8	6.4 ± 2.6	6.4 ± 2.6	0.78
Length of stay (days) (Mean ± SD)	5.0 ± 4.5	4.9 ± 4.5	4.9 ± 4.5	0.89
No. of hospitalization last year (Mean ± SD)	2.2 ± 1.8	1.8 ± 1.6	1.9 ± 1.7	0.16
No. of visits to family doctor 3 months before hospitalization	7.1 ± 7.2	6.1 ± 9.5	6.2 ± 9.1	0.47
No. of visits to specialist doctors last year (Mean ± SD)	3.5 ± 4.5	2.5 ± 3.1	2.7 ± 3.4	0.15
No. of weekly phone calls (< 7 a week)(%)	60.4%	34%	38.9%	0.001
No. of visits (Mean ± SD)	4.6 ± 3.9	5.8 ± 3.6	5.6 ± 3.7	0.03
Functional status: Total Barthel Index (Mean ± SD)	86.8 ± 20	93.5 ± 14.	92 ± 16	0.03
Depression (GDS) (Mean ± SD)	7.1 ± 3.7	5.6 ± 3.2	5.8 ± 3.3	0.005
Cognitive function (MMSE) (Mean ± SE)*	25.1 ± 4.8	27.7 ± 3.2	27.2 ± 3.7	<0.001

Examination of social support variables revealed statistically significant differences between the two groups. Subjects at risk for malnutrition had a higher frequency of phone calls and visitors compared to the malnourished group.

No difference was detected in biochemical measurements – total lymphocyte count (TLC), hemoglobin, WBC, total cholesterol, and transferrin – except for serum albumin. Malnourished participants had a trend of lower serum albumin concentration compared with those at risk for malnutrition (p = 0.06).

**Figure 2:**
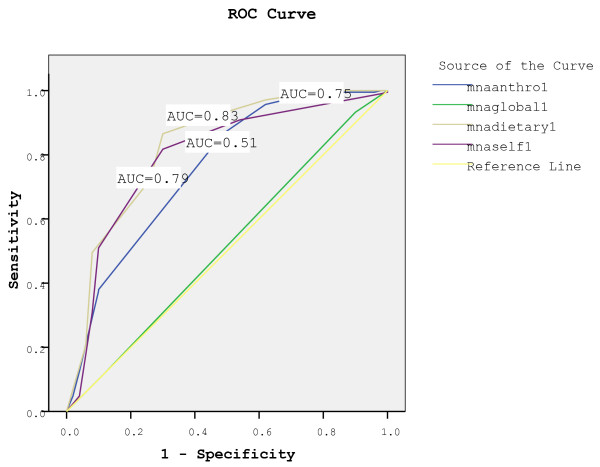
A Receiver Operating Characteristic curve (ROC) for anthropometric (mnaanthro1), global (mnaglobal1), dietary (mnadietary1), and self-assessment of nutritional status (mnaself1) using data from the full version of the MNA.

In order to determine the sources of the difference in nutritional status between the groups, the relationship between Nutritional Status and grouped Mini Nutritional Assessment items were assessed and described in Figure 2. Items were grouped according to the content of the questions, as described in the methods section into MNA anthro-1 that represents the anthropometric assessment, MNAglobal-1 that represents global assessment, MNAdietary-1 that represents dietary assessment questions, and MNAself-1 that represents self-assessment of nutritional and health status. The ROC curve (Figure 2) demonstrates that the dietary assessment part within the MNA is associated with greater area under the curve (AUC) (0.83, p < 0.01) than the anthropometric assessment (0.75, p < 0.01), the global assessment part (0.51, p = 0.75), and the self-assessment of nutritional status (0.79, p < 0.01). Within the dietary assessment part, the prevalence of severe decrease in appetite was significantly higher among the malnourished group (16.7% vs. 8.5% among the at risk population).

Items of the NRI questionnaire were compared and described in Table 2. In the whole group, almost 73% suffered from constipation or diarrhea. Chewing was a problem for 23.3%, swallowing was a problem for 11.6%, vomiting for 18.6%, and nausea for 31% of participants. Almost 74% among the study participants reported that they are on a special diet (Table 2). The malnourished group suffered from more chewing (41.7% vs. 19%), vomiting (31.3% vs. 15.7%), and nausea problems (47.9% vs. 27.1%) compared with those at risk of malnutrition. The at risk group had significantly more artificial teeth compared with the malnourished group.

**Table 2:** Relationship between Nutritional Status and selected items from Nutritional Risk Index (NRI)

Variable	Malnourished	Risk of malnutrition	Total	P value
Constipation/diarrhea	79.2%	71.4%	72.9%	0.28
Artificial teeth	64.6%	79%	76.4%	0.03
Chewing problems	41.7%	19%	23.3%	0.001
Swallowing problems at least 3 times last month	18.8%	10%	11.6%	0.09
Vomiting at least 3 times last month	31.3%	15.7%	18.6%	0.01
Nausea at least 3 times in the last month	47.9%	27.1%	31%	0.005
Are you on a special diet?	75%	73.7%	73.9%	0.85

The independent effect of individual health status and functional and dietary habit variables on nutritional risk were evaluated in a multivariate model (Table 3). The model included variables that were significantly associated with nutritional status in the univariate analyses including family status, depression level, and cognitive and physical functioning. Lower cognitive function (OR = 1.1) and <12 years of education (OR = 3.2) were both risk factors for malnutrition, while lack of chewing problem (OR = 0.3) was protective.

**Table 3:** A Logistic Regression Model to Predict Malnutrition

Variable	OR	*p *value	95% CI
Education ≤12 years	3.22	0.029	1.13–9.19
Family status (being married)	0.75	0.13	0.52–1.09
Total Barthel Index	1.02	0.14	0.99–1.04
Depression (GDS)	0.99	0.82	0.88–1.11
Cognitive function (MMSE)	1.12	0.013	1.02–1.22
Chewing problems	0.35	0.005	0.17–0.72

## Discussion

The main objective of the current study was to characterize malnourished and undernourished elderly people admitted to an acute care ward and particularly to identify risk factors that could be the target for future intervention programs. Based on the MNA evaluation, we showed that 81.5% of the participants of this study were at risk for malnutrition and 18.5% were malnourished. Malnourished participants were less educated, had more depressive symptoms, and lower cognitive and functional status compared with participants at nutritional risk. Moreover, malnourished participants had fewer social contacts including visits and phone calls. The following problems were found to have a significantly higher prevalence among malnourished patients: chewing problems, nausea, and vomiting. These findings indicate the severe impact of these factors on the development of actual malnutrition.

In a study that was conducted among subacute care patients in St. Louis, the prevalence of undernutrition was evaluated, using the MNA. Among 837 patients consecutively admitted during 14 month, the prevalence of malnutrition was 28.8% and 62.5% were at 'nutritional risk' [19]. In another group of very old hospitalized patients (mean age 84.8 ± 8.1 y), 33.2% were at risk for malnutrition and 49.4% were malnourished [21]. In institutionalized women in Spain the prevalence of malnutrition was 7.9% and 61.8% were at risk for malnutrition [38]. In our group, since we used participants who were already screened for nutritional risk and malnutrition the rates were different although it is quite clear that the rate of malnutrition is relatively low. The differences observed may reflect the type of elderly people being screened in each study.

The MNA is a dietary assessment tool that was validated in many different populations [11,32,33] and was shown to be related to several outcomes including mortality, length of hospitalization and complications [19-21]. In a study that assessed the impact of nutritional status measured by the MNA on pressure sores, the MNA provided advantages over using visceral proteins in screening [20]. In our study the laboratory measurements were not related to the MNA results except for serum albumin which was slightly lower among the malnourished group. It is likely that poor nutrition takes considerable lag time until it is manifested in laboratory measurements. The decline of serum albumin is certainly a late phenomenon in terms of malnutrition. Additionally, albumin is a negative acute phase reactant which would likely be diminished in many hospitalized patients who do not suffer from malnutrition. Therefore its futility as an indicator of nutritional status is limited in this scenario.

Among the demographic parameters, we used country of origin as an important parameter. Israel is a multiethnic country with ongoing waves of immigration from various countries: 63.7% of the study population immigrated from Europe/America. The highest percent of malnourished participants immigrated from Africa/Asia. It is our assumption that the high prevalence of malnutrition in this population may stem from the poor living conditions and lower socioeconomic status (SES) highly prevalent in this population throughout their first years in Israel. Therefore, their retirement income is, on average, lower than people who emigrated from European/American countries. Data from the Central Bureau of Statistics indicate that older adults who emigrated from European/American countries are more educated and their retirement income is higher, compared with immigrants from African and Asian countries [39].

Over 64% of the malnourished participants were widowed and over 39.6% were living alone. Marital status and social isolation, especially when combined with recent bereavement or poor social support, have been shown in previous studies to be major risk factors for malnutrition [40,41]. In a case control study comparing health and nutritional values between widowed and married participants, Rosenbloom [42] described reported lack of appetite as an important parameter associated with depression and weight loss in widowed elderly people. In another case control study [40], widowed community dwelling participants lost significantly more weight compared with a control married group. Poor appetite was a significant risk factor for nutritional deterioration [40].

Poor appetite is an important risk factor for nutritional risk. Payette et al. [43], who evaluated a community living elderly population, showed that reported good appetite appeared to be a significant predictor for dietary intake of calories (p < .01) and protein (p < .05). In a previous study by Shahar et al. [44], approximately 20% of the participants reported lack of appetite, or high frequency of feeling no wish to eat. These subjects had lower energy intake as well as lower intake of other nutrients, and thus were considered at risk for nutritional deterioration. In our study, severe loss of appetite was associated with malnutrition. Among the malnourished group, the prevalence of severe loss of appetite was significantly higher (16.7% vs. 8.5%).

The dietary assessment part of the MNA includes questions regarding protein, vegetable and fruit intake, appetite, fluid intake, and difficulties in eating. This part of the assessment has the highest sensitivity and specificity as indicated in the ROC curve. This further highlights the importance of dietary assessment as a mean of detecting nutritional risk.

Depression is the most common cause of unintentional weight loss and under-nutrition in older adults [45,46]. Depression in the elderly is a frequent, treatable, but under-recognized and under-treated, disorder. Patients with depressive symptoms are not identified and thus are seldom treated for this condition [47].

In a retrospective chart review to determine the cause of weight loss in nursing home residents, Morley and Kraenzle [48] also concluded that depression was the most common cause for weight loss. In our study the average number of depressive symptoms was significantly higher among the malnourished participants. We also found that in the malnourished group 81.3% reported weight loss compared to 50.7% among the at risk group.

Nutritional risk is related to functional status [48,49]. Our results indicate that the malnourished group suffered from more functional disabilities according to the Barthel Index and had a higher prevalence of impaired mobility. This observation, however, does not provide conclusive evidence regarding the causal relationship between ADL dependency and malnutrition, since each of these may be the cause of the other.

We did not find any difference in health status between the groups as measured by number of prescribed medications, number of hospitalizations during the year prior to the study, number of family or specialist physician visits, and duration of hospitalization, between the malnourished and at risk groups. However, subjective health evaluation compared with peers was significantly poorer among the malnourished group. The difference in subjective health evaluation may indicate a difference in severity of the disease. Subjective health evaluation in the elderly is considered one of the most accurate measures of health status; its association with malnutrition indicates a close relationship between health and nutritional status.

The malnourished group suffered significantly more from chewing problems, vomiting, and nausea compared to the at risk group. Eating problems and their relation to nutritional status clearly revealed the importance of identifying special problems related to eating and digestion. Mowe et al. showed that chewing problems can lead to a reduced dietary intake and thus to poor nutritional status [49]. Therefore, these problems need to be given closer attention in patient care because of their cumulative effect on dietary intake. Earlier identification of these risk factors may allow a more efficacious intervention which may prevent actual malnutrition from occurring.

Our study suffers from several limitations. The study examined a selected population of hospitalized elderly patients at risk for malnutrition; however, characterization of these groups and the distinction between the levels of undernutrition is important for developing targeted interventions. In addition, the study is a cross-sectional survey and thus cannot serve to determine temporal relationships.

Our study evaluated the association between in-hospital malnutrition and several risk parameters. Of all the parameters studied, the difficulty in consuming foods was found to be highly associated with the development of malnutrition. The most important predictors of actual malnutrition in these patients were lower education, poorer cognitive status, and chewing problems. At least some of these parameters are amenable to pharmacological and non-pharmacological treatment modalities. Therefore, given the critical importance of nutritional status in the hospitalized elderly and its impact on mortality and morbidity [19-21], an emphasis should be placed on correcting these problems. An example of such interventions may be withholding medications, performing speech therapy evaluation, or naturally changing food texture and constituents. We feel that our findings highlight the need for a nutritional intervention trial among at risk and malnourished hospitalized patients.

## Competing interests

The author(s) declare that they have no competing interests.

## Authors' contributions

IF-Main writer and research coordinator

LG-Data collection, study design and writing

HC-Medical counseling, collection and evaluating medical information of the patients

IH-Medical counseling, collection and evaluating medical information of the patients

NB-Data collection, statistical analyses and writing

ME-Medical counseling, collecting and evaluation of medical records in internal medicine department F

DF-Principle investigator, statistical analyses and writing

DS-Principle investigator, study design and writing

All authors read and approved the final manuscript
